# Courtship behaviour of *Phlebotomus papatasi* the sand fly vector of cutaneous leishmaniasis

**DOI:** 10.1186/1756-3305-5-179

**Published:** 2012-08-30

**Authors:** Ifhem Chelbi, DP Bray, JGC Hamilton

**Affiliations:** 1 Chemical Ecology Group, Institute for Science and Technology in Medicine, Keele University, ST5 5BG, Keele, UK; 2 Laboratoire d’Ecologie des Systèmes Vectoriels, Institut Pasteur de Tunis, 13 Place Pasteur, 1002, Tunis, Tunisia

**Keywords:** *Phlebotomus papatasi*, *Leishmania major*, Mating behaviour, Kinetogram, Abdomen bending, Pheromone

## Abstract

**Background:**

The sand fly *Phlebotomus papatasi* is an Old World vector of *Leishmania major*, the etiologic agent of zoonotic cutaneous leishmaniasis. This study describes the courtship behaviour of *P. papatasi* and compares it with that of *Lutzomyia longipalpis*, the New World vector of visceral leishmaniasis. Understanding the details of courtship behaviour in *P. papatasi* may help us to understand the role of sex pheromones in this important vector.

**Results:**

*P. papatasi* courtship was found to start with the female touching the male, leading him to begin abdomen bending and wing flapping. Following a period of leg rubbing and facing, the male flaps his wings while approaching the female. The female then briefly flaps her wings in response, to indicate that she is willing to mate, thereby signaling the male to begin copulation. Male *P. papatasi* did not engage in parading behaviour, which is performed by male *L. longipalpis* to mark out individual territories during lekking (the establishment and maintenance of mating aggregations), or wing-flap during copulation, believed to function in the production of audio signals important to mate recognition. In *P. papatasi* the only predictor of mating success for males was previous copulation attempts and for females stationary wing-flapping. By contrast, male *L. longipalpis* mating success is predicted by male approach-flapping and semi-circling behaviour and for females stationary wing-flapping.

**Conclusions:**

The results show that there are important differences between the mating behaviours of *P. papatasi* and *L. longipalpis*. Abdomen bending, which does not occur in *L. longipalpis*, may act in the release of sex pheromone from an as yet unidentified site in the male abdomen. In male *L. longipalpis* wing-flapping is believed to be associated with distribution of male pheromone. These different behaviours are likely to signify significant differences in how pheromone is used, an observation that is consistent with field and laboratory observations.

## Background

The sand fly *Phlebotomus papatasi* (Diptera: Psychodidae) is the principle vector of *Leishmania major* (Kinetoplastida: Trypanosomatidae), the etiologic agent of zoonotic cutaneous leishmaniasis (ZCL) in the Old World [1]. Transmission occurs because of the blood-feeding behaviour of female *P. papatasi* on infected animals and human hosts. There is no vaccine to protect against this disfiguring disease [2], and treatment is prolonged, unpleasant and expensive [3]. * P. papatasi * also transmits a number of viruses recognized as neglected human pathogens [4].

Traditionally, efforts to control *P. papatasi* have relied on killing sand flies by treating resting sites and animal burrows with insecticides [5]. However, in addition to environmental concerns, insecticide spraying is expensive and difficult to maintain effectively over long periods [6] and can result in the development of tolerance or resistance [5]. A number of alternatives to insecticide use have been tried experimentally e.g. insecticide impregnated dog collars [7], toxic sugar baits [8] and bed nets [9]. However, none of these approaches have been widely adopted, and no single solution is likely to be effective in controlling *P. papatasi* across its entire geographic range, which encompasses parts of Africa, India, Asia and Europe [10]. The vector, and the diseases it transmits, may become further widespread because of man-made and other local and global environmental changes [4,11,12].

In common with many sand fly vectors of leishmaniasis, studies of behaviour in *P. papatasi* have been very limited. This lack of fundamental knowledge is a barrier which must be overcome if new, more effective control measures are to be developed. In particular, we know very little about courtship and mating in this species. Courtship is a key determinant of reproductive success, and as such represents a major target for disruption and exploitation as a means of controlling populations of insect pests and vectors e.g. through use of synthetic sex pheromones [13].

To date, studies of reproductive behaviour in *P. papatasi* have focused almost exclusively on the role of blood-feeding in successful reproduction. While females of some populations require a blood meal in order to produce eggs, others do not [14-16] and the factors controlling autogenic development remain unclear. Timing of mating seems to be independent of blood-feeding [17], although presence of a blood meal in the female may be necessary in some strains to allow sperm to travel towards the spermathecae and subsequently fertilize eggs [18]. Observational evidence of mating and examination of spermathecae also suggests that, unlike the New World leishmaniasis vector, *L. longipalpis*, female *P. papatasi* may mate with more than one male per gonadotropic cycle, providing opportunities for post-copulatory sexual selection [18,19]. What determines mating success in *P. papatasi* is currently unknown, although recent evidence suggests that male-produced pheromones play a role in attracting females at a distance [20]. Understanding the behavioural context in which these chemical signals are used will be important if they are to be exploited in future control programmes.

The aim of this study was to provide the first quantitative description of courtship in *P. papatasi*, through careful observation of male–female pairs under laboratory conditions. Particular emphasis was given to comparing mating behaviour of *P. papatasi* with that of *L. longipalpis*, the principle vector of visceral leishmaniasis in the New World [21], which has been studied in much greater detail [22,23], and for which pheromone-based control strategies are being developed [24,25].

We began by describing and quantifying individual male and female behaviours performed during courtship, and comparing them to those reported previously from studies of *L. longipalpis* and other species of sand fly [26]. We then determined how males and females respond to one another during courtship, and the sequence of events which occurs prior to copulation, by using a sequential analysis technique [27,28] applied previously to the description of mating in * Drosophila *[29,30] and * L. longipalpis *[22]. Finally, we identified those behaviours that are most important in determining whether courtship proceeds to copulation, and may therefore be associated with signals critical to mating success.

## Methods

### Sand fly rearing

Sand flies were from a laboratory colony of *P. papatasi* established from adults collected in El Felta, Sidi Bouzid, Tunisia (34° 5’ N, 9° 29’ E) in 2009, and maintained at Keele University over 4 – 5 generations and were not infected with leishmania parasites [14]. Adults were kept in Barraud cages at 27 °C 95% RH under a 12:12 (L:D) photocycle. Females were blood fed 3 days post emergence in accordance with UK Home Office Licence requirements.

All flies used in observations were placed into single-sex cages within 7 h of eclosion (prior to rotation of male genitalia) to prevent mating prior to trials.

### Recording of courtship behaviour

Observations of interactions between pairs of male and female *P. papatasi* were conducted using a courtship arena consisting of a 15 mm high, 22 mm diameter plastic dish lined with plaster of Paris on the internal vertical surface and base. The top of the arena was covered with a transparent plastic lid to prevent flies from escaping, while facilitating videoing of courtship behaviour from above. All observations were conducted under white fluorescent light in a temperature controlled (28°C ± 1°C) bioassay room, with additional illumination for filming provided by a fibre-optic light source (KL 500; Schott UK Ltd, Stafford, UK).

Courtship behaviour was recorded using a colour video camera (TK-1280E; JVC, London, UK) fitted with a zoom lens (Computar 18–108 mm, f 2.5 manual focus; CBC (Europe) Ltd, London, UK) and supported 30 cm above the courtship arena using a copy stand (CS-920; Tracksys Ltd, Nottingham, UK). Output from the camera was fed through a vertical interval time code (VITC) generator (AEC-BOX-18; Adrienne Electronic Corp, Las Vegas, NV, USA) to a time-lapse security video recorder (VCR) (HS1024; Mitsubishi Electric, Hatfield, UK) set to continuous recording. A feed from the VCR was also sent to a colour monitor (Triniton KV-14M1U; Sony, Thatcham, UK) to facilitate camera adjustments and to view observations while filming.

For each observation, a male sand fly was first placed into the courtship arena via a small hole in the transparent plastic cover using an aspirator. After 5 min the VCR was set to record, and a female placed into the arena using the aspirator. Trials ended once flies had separated post-copulation, or after 30 min where copulation did not occur. Recordings of 30 different pairs were made in total, using a clean arena and lid for each observation.

### Quantitative analysis of courtship behaviour

Behaviours of male and female *P. papatasi* observed during courtship were coded into mutually exclusive categories (Table 1), using a *P. papatasi*-specific adaptation of the scheme previously derived from examination of * L. longipalpis *[22]. The order and occurrence of these behaviours during trials was then quantified using the Observer Base Package for DOS and Support Package for Video Tape Analysis (Version 3.1; Noldus Information Technology, Wageningen, the Netherlands). This software allowed video recordings to be observed in slow motion, while occurrence of individual behaviours was recorded onto the computer through a series of key presses. The resultant data could then be analyzed to determine the length and frequency of each individual behaviour, as well as the order in which they occurred. Key presses were synchronized to the behaviour observed on tape by connecting the VCR output to a PC-VITC card (Adrienne Electronic Corp.) which relayed the time code imprinted by the VITC generator during recording to the Observer software on playback. 

**Table 1:** **Behaviours performed during *P. papatasi *courtship**

	**Name of behaviour**	**Description**
**Male and female behaviours**		
1	*Not courting*	Sand fly stationary or moving around the arena, but not interacting with courtship partner.
2	*Facing*	Male and female stand facing, slightly adjacent to one other, approximately one body length apart.
3	*Leg Rubbing*	Sand fly appears to cross the tips of the front legs while rubbing them against one another. Contact may also be made with the mouth parts and antennae.
4	*Touching*	Sand fly touches partner, usually making contact with the tips of the legs or antennae. Females were also observed to touch the male abdomen at the start of courtship.
5	*Stationary wing-flapping*	Sand fly remains stationary while beating both wings simultaneously in a series of single flaps, rotating both wings forward in an arc up to a maximum of 70° from the abdomen.
6	*Copulation*	Male and female copulate facing in opposite directions, with the tips of the abdomen joined. Both male and female remain motionless throughout.
**Male-only behaviours**		
7	*Abdomen bending*	Male bends his abdomen to the left and right, swaying the genitalia in an arc between the rear legs. Often interspersed with brief displays of wing-flapping.
8	*Approach-flapping*	Male alternates between stepping towards the female and wing-flapping, often as a precursor to copulation.
9	*Copulation attempt*	Male bends abdomen forward towards the head before briefly taking off and attempting to make genital contact with the female.

Differences between males and females in the frequency or time spent performing individual behaviours during observations were examined using Wilcoxon’s signed rank test. Comparisons of duration of individual behaviours performed by males and females were performed using Welch’s *t*-test, which does not assume equal variances between groups [31].

### Analysis of interactions during courtship

The sequence of behaviours occurring during courtship was analyzed through logit-linear modeling [27,28] in PASW (SPSS) v18 (IBM Corp, New York, USA). For each observation, the frequency of changes from one courtship behaviour to another (e.g., male wing-flapping followed by female touching) was calculated, ignoring periods of ‘not courting’ between behaviours. The resultant transition matrices for each observation were then summed to create a total transition matrix (Table 2), which was then divided into 4 sub-matrices (male-male behaviours, female-female behaviours, male–female behaviours, and female–male behaviours), prior to statistical analysis. Facing, performed simultaneously by both sexes, was included as both a male and female behaviour. 

**Table 2:** **Number of transitions from preceding to following behaviours during *Phlebotomus papatasi *courtship**

		**Following behaviour**			
		**Female behaviours**			
**Preceding behaviour**		**facing**	**leg rubbing**	**touching**	**stationary wing-flapping**
		Female behaviours					
	facing	2†	0†	15†	0†	
	leg rubbing	0†	0†	7†	1†	
	touching	6†	4†	94†	4†	
	stationary wing- flapping	0†	2†	2†	0†	
		Male behaviours					
						
	abdomen bend	3	3	65*	0	
	approach-flapping	0	0	2	6*	
	copulation attempt	0	0	1	0	
	facing	2	0	15	0	
	leg rubbing	3*	0	5	0	
	touching	13*	2	21	4	
	stationary wing- flapping	3	3	27	5	

* Significant positive transition (*P* < 0.05).

† Significance of individual transition not assessed (see text for details).

For each of the four sub-matrices, we first tested whether overall following behaviour was influenced by preceding behaviour (for example, whether female behaviour in general was influenced by the preceding behaviour of the male), before examining whether individual behavioural transitions (e.g. male stationary wing-flapping followed by female touching) occurred more often than expected by chance. This initial omnibus test was performed by entering ‘following’ behaviour as the dependent variable in a logit-linear model with ‘preceding behaviour’ entered as the independent variable. Whether preceding behaviour significantly (*P* < 0.05) affected following behaviour was assessed through the log-likelihood goodness of fit statistic (G) [28], following deletion of preceding behaviour from the model.

**Figure 1:**
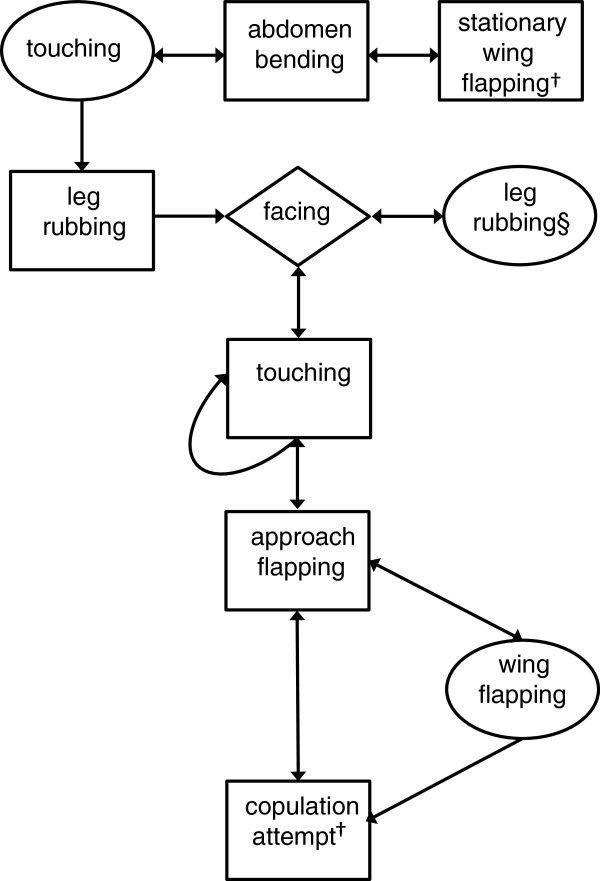
**Kinetogram of* P. papatasi *courtship derived from analysis of 30 male–female pairs showing statistically significant transitions.** Rectangles indicate male behaviours, ovals female behaviours, and diamonds joint male–female behaviours. † Behaviour occurs significantly more frequently in successful courtships. § Behaviour occurs significantly more frequently in unsuccessful courtships.

Where a significant association between preceding and following behaviour was found, the significance of individual behavioural transitions was then assessed through examination of residuals following deletion of preceding behaviour in the model. Adjusted residuals >1.96 indicated sequences of behaviour occurring significantly (*P* < 0.05) more often than expected by chance alone [27]. These significant individual transitions were then used to build a kinetogram (Figure 1) illustrating the overall sequence of behaviours occurring during courtship. 

### Identification of courtship behaviours predicting copulation success

Fisher’s exact test was used to determine which male and female behaviours occurred more frequently in courtships leading to copulation than unsuccessful courtships, and might therefore be most crucial to mating success.

## Results

### Individual behaviours performed during courtship

*P. papatasi* courtship consisted of a number of distinct male and female behaviours (Table 1), separated by periods of remaining stationary or moving around the arena (‘not courting’). In total, males spent a significantly greater percentage of time (excluding the time spent copulating) actively ‘courting’ than females (males 20.9% ± 3.1; females: 4.9% ± 1.0, W = 15, N = 30, *P* < 0.001).

Both males and females performed stationary wing-flapping (Table 1: behaviour 5). Males spent more time stationary wing-flapping during courtship than females (mean time (s): males 40.6 ± 11.1; females 1.7 ± 0.4, W = 28, N = 24, *P* < 0.001). They also stationary wing-flapped more frequently during courtship (mean frequency: males 6.0 ± 1.7; females 0.7 ± 0.2, W = 9, N = 20, *P* < 0.001). Individual male stationary wing-flapping displays also lasted longer than those of the females (mean duration (s): males 6.8 ± 0.7; females 2.3 ± 0.4, t = 5.8, df = 172, *P* < 0.001), which were usually restricted to a few individual wing beats, normally occurring shortly prior to a copulation attempt.

Both sexes also engaged in touching (behaviour 4), with contact most often made with the legs or antennae. Females were observed to touch the male abdomen at the start of courtship, and there was also a tendency toward females initiating touching more frequently than males (mean frequency: males 4.7 ± 0.8, females 8.3 ± 1.5, W = 117, N = 28, P < 0.06).

Both males and females occasionally performed a solitary behaviour classified as leg rubbing (behaviour 3). This involved rubbing the front legs against one another, with contact possibly also made with the mouth parts. There was no significant difference between males and females in the frequency of this behaviour (mean frequency per trial: males 0.6 ± 0.1; females 0.5 ± 0.2, W = 45.5, N = 14, NS) or time spent leg rubbing (mean time (s): males 21.7 ± 5.5; females 17.8 ± 4.9 W = 83, N = 19, NS).

Facing, (behaviour 2) which was performed simultaneously by males and females, occurred between bouts of more active courting. It was noticeable by the manner in which males and females turned towards each other and remained motionless for several seconds (mean frequency per trial: 0.9 ± 0.2; mean duration (s): 21.5 ± 11.8).

Three behaviours, abdomen bending (behaviour 7), approach flapping (behaviour 8) and attempted copulation (behaviour 9) were performed exclusively by males. Abdomen bending (mean frequency per trial: 8.1 ± 1.9, mean duration (s): 18.1 ± 2.3), was performed in bursts between periods of wing flapping. Approach flapping (mean frequency per trial 1.7 ± 0.3, mean duration (s): 6.0 ± 0.89) combined walking towards the female and wing flapping, and often preceded attempted copulation (mean frequency per trial: 0.9 ± 0.2, mean duration (s) 2.2 ± 1.0).

In total, 14 out of 30 (47%) of the courtships that were observed proceeded to copulation. Where successful, males achieved copulation on their first (n = 11), second (n = 2) or third (n = 1) attempt. Courtship duration (copulation latency), measured from the time when the female entered the arena in which the male was already present, varied between 96.4 s (1.6 minutes) to 1308 s (21.8 min) (mean duration (s) 630.2 ± 95.6). Where courtship was successful, copulation lasted between 1431.2 s (23.9 min) and 2426.4 s (40.4 min) (mean duration (s): 1914.4 ± 69.4).

### Sequence of behaviours during courtship

Overall, there was a significant effect of preceding behaviour on following behaviour in male-male transitions (G = 258.6, df = 36, *P* < 0.001), with a number of significant behavioural sequences identified (Table 2). However, no such association was found for female-female transitions (G = 12.65, df = 9, *P* = 0.18).

Significant effects of preceding behaviour were found overall both for male–female transitions (G = 66.1, df = 18, *P* < 0.001), and female–male transitions (G = 72.1, df = 18, *P* < 0.001), with several significant behavioural sequences identified between the sexes (Table 2).

The kinetogram (Figure 1), which was derived from Table 2 suggests the following simplified model of *P. papatasi* courtship behaviour. Courtship is initiated with the female touching the male abdomen, leading him to begin abdomen bending. The male then rubs his front legs together, before male and female enter into a period of facing one another. The male then flaps his wings (stationary-flapping), and either returns to abdomen bending, or approaches the female while wing flapping (approach-flapping). This leads the female herself to flap her wings, and the male to attempt copulation. In addition, inspection of the kinetogram also suggests that female leg rubbing also leads to the male touching the female, which also precedes attempted copulation.

### Behaviours predicting copulation

The only male behaviour whose occurrence was found to predict successful courtship was attempted copulation (*P* < 0.001, Table 3). Males attempting to copulate was a prerequisite of mating, and males did not attempt to copulate in 12 out of 16 (75%) of unsuccessful trials. Successful copulation was not predicted by occurrence of male stationary wing-flapping, approach-flapping, touching, leg rubbing or abdomen bending (Table 3).

**Table 3:** Occurrence of individual behaviours in successful and unsuccessful courtships

		**Unsuccessful courtships (N=16)**	**Successful courtships (n=14)**
			
		Male behaviours	
	abdomen bending	14 (88%)	10 (71%)
	approach flapping	11 (69%)	12 (86%)
	copulation attempt†**	4 (25%)	14 (100%)
	facing	10 (63%)	5 (36%)
	leg rubbing	9 (56%)	4 (29%)
	touching	15 (94%)	13 (93%)
	stationary wing- flapping	16 (100%)	14 (100%)
		Female behaviours	
			
	facing	10 (63%)	5 (36%)
	leg rubbing§*	10 (63%)	1 (7%)
	touching	14 (88%)	10 (71%)
	stationary wing- flapping†*	4 (25%)	11 (79%)

† Behaviour more likely to occur in successful courtships (Fisher’s exact test).

§ Behaviour more likely to occur in unsuccessful courtships.

* *P*<0.05, ** *P*<0.01, *** *P*<0.001.

Female wing flapping occurred significantly more often in courtships that concluded with copulation (*P* < 0.01, Table 3), and occurred in 79% of successful courtships, and only 25% of unsuccessful courtships. Interestingly, copulation was also more likely to take place when female leg rubbing did not occur (*P* < 0.01, Table 3). Courtship success was not influenced by the occurrence of female touching, or facing behaviour (Table 3).

## Discussion

Courtship in *P. papatasi* consisted of a number of male and female behaviours, some of which are similar to those previously reported from other sand fly species. As in *L. longipalpis*, touching between male and female was a common occurrence in *P. papatasi* courtship, with contact most often made with the tips of the legs or the antennae. The function of this behaviour is unknown, but has been hypothesized to represent the transfer and reception of cuticular hydrocarbons [22], important chemical signals in the mating behaviour of a number of insect species [32]. Presence of cuticular hydrocarbons has been reported from a number of * Phlebotomus * species, including *P. papatasi*[33] with differences in hydrocarbon profiles found between species, populations and sexes [33-36]. It is therefore quite conceivable that *P. papatasi* use this information in recognizing, and perhaps discriminating between, potential mates.

Female touching at the start of courtship was found to lead males to initiate abdomen bending, a display behaviour involving repeated lateral bending of the abdomen interspersed with periods of wing flapping (Additional file 1). Similar abdomen bending behaviour has been described in male sand flies from both the Old and New World [26], but the exact purpose of this behaviour is unknown. Both field and laboratory experiments indicate that male *P. papatasi* produce a sex pheromone to attract females [20]. Although the site of production of these chemicals has yet to be determined, abdomen bending might facilitate the release of sex pheromone from abdominal tergites. This behaviour invites further investigation. If abdomen bending does function as suggested, it may indicate the presence of sex pheromones in a range of sandfly species, including *L. vexator* in the United States [37], * P. longipes * from Ethiopia [38] and *P. martini* from Kenya [39] all of which exhibit abdomen bending behaviour.

Both male and female *P. papatasi* were observed to engage in a behaviour which (when viewed from above), appeared to show individuals rubbing their front legs together, with contact perhaps also made with the mouth parts and antennae. Close up recordings from different angles will be required to ascertain the exact nature of this behaviour, and at this stage we can only speculate on its function. Interestingly, female leg rubbing was found to occur more often in courtships not leading to copulation, and as such perhaps indicates when females are unreceptive to mating. If contact with the mouth parts is made, it might allow the transfer of female aggregation pheromone [40] from their site of production on the palps to the legs, which could signal to males the intention to blood feed, rather than to mate.

Leg rubbing preceded a period of facing, in which both sexes stood apparently motionless for a period of several minutes. Such apparent waiting behaviour halfway through courtship might indicate the need for a physiological change to occur in either partner prior to mating, or time during which a courtship signal is produced that is not detected through our video analysis.

As reported in * L. longipalpis *[22], length of copulation varied considerably between pairs of *P. papatasi*, and at maximum exceeded forty minutes. The extent to which length of copulation is linked to reproductive success in sand flies is unknown, although much shorter copulations have been reported from studies in which female *P. papatasi* may have been provided with a choice of more than one potential mate [16,18]. Males and females copulated back-to-back as previously described [41] and as occurs in *L. longipalpis*[22,42]. * P. papatasi * did not engage in ‘piggy backing’, a behaviour observed in * P. dubosci *[43] which may function in mate recognition or mate guarding.

Wing-flapping is a common behaviour in sand fly courtship, and has been reported from observations of mating in species from both Old and New World genera [26]. * In P. papatasi *, as in *L. longipalpis*, wing-flapping was performed by both sexes: males flapped their wings while approaching the female, triggering brief wing-flapping displays by the female, which, as in *L. longipalpis*, predicted the onset of copulation [22]. The modality through which wing-flapping signals communicate in *P. papatasi* is unknown: in *L. longipalpis*, it has been hypothesized that male wing-flapping functions to disperse sex pheromone [23,44,45], and it could conceivably have a similar function in *P. papatasi*, directing pheromone released through abdomen bending towards potential mates. Considerable work will be needed however, to test this hypothesis, including the identification of the putative male pheromone, and its site of production. As there is no evidence of pheromone production in female *L. longipalpis* or *P. papatasi*, the brief wing-flapping displays presumably communicate through production of visual or auditory signals.

In *L. longipalpis*, males flap their wings during copulation to produce auditory signals believed to function in mate recognition, with different sibling species within the *L. longipalpis* complex producing signals with distinct audio characteristics [46,47]. These copulatory signals are not restricted to *L. longipalpis*, and have recently been recorded from * L. cruzi *[48], * L. migonei *[49], and *L. intermedia*[50]. Here, male *P. papatasi* only flapped their wings briefly and infrequently at the start of copulation. If this does result in an audio signal, it would most likely be considerably less complex than those produced by *Lutzomyia spp.*

Genetic analyses indicates that, in comparison to *L. longipalpisP. papatasi* is genetically homogeneous throughout its geographical range [10,51,52] although other evidence suggests a more heterogenous population substructure [53]. If all *P. papatasi* are reproductively compatible, they probably produce similar signals and there would be no requirement for complex audio signals during copulation, or for populations to produce different sex pheromones, as occurs in * L. longipalpis *[54]. Wing-flapping at the start of copulation in *P. papatasi* could therefore function simply in the dispersal of pheromone towards the female during mating, perhaps as an arrestant, or to assist the male in manoeuvring the aedeagal filaments closer to the female spermathecal ducts prior to the deposition of spermatophores [41].

Previous descriptions of mating behaviour in *L. longipalpis* have described how courting males mark out individual territories through ‘parading’, a behaviour comprising a distinctive three-beat walk, combined with simultaneous wing flapping [22,42]. Here, *P. papatasi* were found only to wing flap either when stationary in conjunction with abdomen bending (after the female made contact with the male abdomen), or while approaching the female prior to copulation. The lack of parading behaviour suggests either that male *P. papatasi* do not defend territories from other males, or that they use different mechanisms for doing so, perhaps only induced by the presence of a rival. Further studies examining male-male interactions will be needed to determine the extent to which male *P. papatasi* ‘compete’ for females, and the behavioural signals involved.

The results of this study add to a growing body of evidence which suggests the mating system of *P. papatasi* is substantially different from that of *L. longipalpis*. In *L. longipalpis*, mating takes place at the same time as blood-feeding, with males forming leks on or near a host animal [42,55]. This strategy of aggregating near a host presumably increases the chances of males encountering females with which to mate [56], as a blood meal is a necessary prerequisite for egg production [57]. Within the lek, females are then free to choose with whom to mate, with a small number of the most attractive males receiving the majority of the matings [42,58]. Male-produced sex pheromones play an important role in several key aspects of this mating system, including mate finding (attracting females to the lek [59,60]), mate recognition (different members of the *L. longipalpis* species complex produce different pheromones; [61] and mate assessment, with females preferring males which produce more pheromone [23]. It is presumably the influence of sexual selection on this latter preference that has driven male *L. longipalpis* to produce large amounts of sex pheromone, which is then stored in the body until needed.

In *P. papatasi*, the association between blood-feeding and mating is less clear. A blood meal is not always a prerequisite for egg production in this species [14-16], and the extent to which mating takes place in aggregations on host animals is unknown. Current evidence may also suggest that male *P. papatasi* do not lek in the same manner as *L. longipalpis*: females are not attracted to large male aggregations [20], and males have not yet been observed to engage in behaviour which might function in defending territories against other males. Furthermore, while there is behavioural evidence to support the existence of male-produced sex pheromones in * P. papatasi *[20], this attractive chemical has not been found stored in large amounts in the body (J.G.C. Hamilton pers. obs.). This may indicate that sex pheromone does not play the same role in mate choice as it does in *L. longipalpis*, and has therefore not been subject to the same directional selection for greater pheromone production. In addition, while male wing flapping has been found to correlate with mating success in * L. longipalpis *[22,23], none of the male behaviours described here were identified as predictors of successful courtship, other than attempting to copulate.

Rather than choosing males through pheromone roduction and wing-flapping, female *P. papatasi* may instead choose with whom to reproduce through post-mating selection. Examination of spermathecae in this species indicates that females can store sperms from a number of males, and may have some choice in which are used in the fertilization of eggs [18]. A mating system in which females mate multiple times and store sperm until needed might suggest that males are encountered less frequently by female *P. papatasi* than in the lekking system of *L. longipalpis*.

This detailed observational study, the first in *P. papatasi*, allowed us to determine and classify a number of behaviours that occur in male–female interactions, and therefore may play a role in courtship. It also allowed us to make comparisons with behaviours observed in the interactions between males and females of the New World vector *L. longipalpis*. Further experimental work, in which these individual behaviours can be manipulated, will be required to determine which of these behaviours (if any) are exclusive to courtship, and their precise function in regulating mating success.

## Conclusions

The courtship behaviour of *P. papatasi* is substantially different to that of *L. longipalpis*. Understanding the wider mating system of *P. papatasi* will be crucial in the development of sex pheromones as tools for control in this species. The ‘lure and kill’ strategy currently being developed against *L. longipalpis* involves release of large quantities of pheromone from a single point, close to an animal host, in order to mimic an aggregation of males with which to attract females to a trap or insecticide. If male *P. papatasi* do not lek on hosts, such a strategy is unlikely to be successful in attracting females of this species. Instead, a mating disruption strategy, releasing synthetic sex pheromone over a wide area to prevent males and females locating one another, may be more effective. Future studies should examine the role of the host in the mating system of *P. papatasi* and the extent to which males form mating aggregations or leks.

## Competing interests

The authors declare that they have no competing interests.

## Authors’ contributions

IC and DPB contributed equally to execution of the study. IC carried out the recordings of the mating interactions and behavioural coding. DPB carried out the design of the study, statistical analysis of behavioural elements and produced the resultant kinetogram. JGCH conceived, contributed to the design and coordinated the study. All three authors contributed to drafting the manuscript and read and approved the final version.

## Supplementary Material

Additional file 1 **Video recording of abdomen bending display behaviour of male *P. papatasi *(bottom right of courtship arena).** Abdomen bending is initiated following female touching of the male abdomen, and is interspersed with periods of male wing flapping. Click here for file
